# Mediating Role of Muscle Mass in the Relationship Between Physical Activity and Bone Mass in Female University Students in China

**DOI:** 10.1155/ppar/5484324

**Published:** 2026-04-03

**Authors:** Jiaye Wang, Zheqing Zhang, Shitian Li, Huan-yu Hu, Haojian Chen, Zhendi Zhang, Xiaocui Lu, Yiran Yan

**Affiliations:** ^1^ School of Public Health, Southern Medical University, Guangzhou City, Guangdong Province, China, fimmu.com; ^2^ Department of Nutrition and Food Hygiene, Guangdong Provincial Key Laboratory of Tropical Disease Research, School of Public Health, Southern Medical University, Guangzhou City, Guangdong Province, China, fimmu.com; ^3^ National Clinical Research Center for Cardiovascular Diseases, Fuwai Hospital, Chinese Academy of Medical Sciences and Peking Union Medical College, National Center for Cardiovascular Disease, Beijing, China, fuwaihospital.org; ^4^ Preventive Medicine Experimental Teaching Center, Guangdong Provincial Key Laboratory of Tropical Disease Research, School of Public Health, Southern Medical University, Guangzhou City, Guangdong Province, China, fimmu.com; ^5^ Department of Traumatic Orthopedics, Orthopedic Medical Center, Zhujiang Hospital, Southern Medical University, Guangzhou City, Guangdong Province, China, fimmu.com

**Keywords:** bone mineral density (BMD), female university student, mediating role, muscle mass, physical activity

## Abstract

**Objective:**

We are aimed at investigating the relationships among muscle mass, physical activity, and calcaneal bone mineral density (BMD) in female university students, and exploring the mediating role of muscle mass in the association between physical activity and calcaneal BMD.

**Methods:**

Using stratified cluster sampling, 593 female university students aged 19–25 of different grades were selected. Anthropocentric characteristics, body composition, and calcaneal BMD were measured. Statistical methods including multiple linear/binary logistic regression models, mediation analyses, sensitivity analyses, and subgroup analysis were performed.

**Results:**

The normal and abnormal BMD groups significantly varied in both total skeletal muscle mass (TSM) and total skeletal muscle mass index (TSMI) (t[TSM] = 3.216, t[TSMI] = 3.304, *p* < 0.01), and also diverse in physical activity (*p* < 0.05). The increment in muscle mass might be related to an increase in BMD (*β*[TSM] = 0.089, *β*[TSMI] = 0.265, *p* < 0.01) and a decrease in the probability of abnormal BMD (*β*[TSM] = −0.193, *β*[TSMI] = −0.682, *p* < 0.01). The effect of physical activity on calcaneal BMD is significant (*β* = −0.109, *p* < 0.05). Muscle mass plays a mediating role in the relationship between physical activity and calcaneal BMD, and the subgroup mediation sensitivity analyses indicated that the mediation effect only in the overweight group had significant mediation effects and relatively high robustness (Rho = −0.2, R^2^_MR^2^_Y = 0.04).

**Conclusion:**

Muscle mass and physical activity both present a significant positive correlation with calcaneal BMD in female university students, and high activity level is a protective factor for abnormal BMD. Muscle mass performs a mediating role in the relationship of physical activity and calcaneal BMD in female university students of the overweight group.

## 1. Introduction

As aging progresses in China, osteoporosis has become a prevalent health concern among older adults, significantly raising the risks of fractures and falls, which subsequently increase disability and mortality rates. [1–4] Bone mineral density (BMD) serves as an important marker of bone mass and a significant indicator for assessing bone health and preventing osteoporosis. [5]

Physical activity (PA) is recognized as one of the key factors influencing BMD. [6–11] As a weight‐bearing site, calcaneal BMD is closely related to overall bone health. [12, 13] Muscle mass, as the immediate target of PA, potentially serves as a mediator in the association between PA and BMD. [14–17] As females generally present with lower BMD, reduced PA, and smaller muscle mass than males, attention to female BMD is undeniably a core concern for clinical and public health domains. [18, 19]

This research focuses on the calcaneal BMD of female university students aged 19–25 at Southern Medical University. By analyzing the relationships among muscle mass, PA, and calcaneal BMD, exploring the mediating effect of muscle mass on the association between PA and bone in female university students, and further exploring the potential confounding factors such as BMI, this research is aimed at providing insight into preventing bone mass reduction and osteoporosis in women.

## 2. Materials and Methods

### 2.1. Subjects

This study employed a stratified cluster sampling method to select female university student volunteers from different academic years at a university as the research subjects. Women were excluded from the study including (a) those who had cardiovascular diseases (e.g., pacemaker implantation), musculoskeletal‐related diseases restricting PA, malignant tumors, autoimmune diseases, metabolic or endocrine disorders, or recent fractures; (b) those who had experienced significant weight fluctuations due to weight loss or gain in a short period; (c) patients who had undergone hormone replacement therapy in the past 6 months; and (d) those who were unwilling to sign the informed consent form.

The sample size was estimated using G∗Power 3.1 for a multiple regression model, with the number of predictors set to 2 (consistent with the mediation pathway). Employing a single mediation model, with a conservative small effect size (f^2^ = 0.02), *α* = 0.05, and power = 0.8), G∗Power calculated a minimum sample size of 465 cases (without considering missing data). Ultimately, taking a 10% missing data rate into account, 518 female university students aged 19–25 years who met the study criteria were enrolled. The Biomedical Ethics Committee of Southern Medical University granted approval for this investigation (approval number: Medical Ethics Review of Southern Medical University [2022] No. 32), and all research adhered to World Health Organization (WHO) (2016) the ethical guidelines for research with human subjects established by the World Medical Association Declaration of Helsinki (1964). [20, 21]

### 2.2. Collection of General Information

Demographic data and lifestyle information were gathered via in‐person interviews employing standardized questionnaires. The interviews were conducted by research team members who had received standardized training. The participants were apprised of the research objective and granted informed consent before data collection commenced. Lifestyle information collected included PA, intake of dairy products, carbonated beverages, and tea, and whether to take calcium supplements.

### 2.3. PA

According to the WHO Guidelines on Physical Activity and Sedentary Behaviour (2020), adults are recommended to accumulate at least 150–300 minutes of moderate‐intensity aerobic physical activity (PA) per week, or an equivalent combination. For the purpose of this study, using 30 min as the minimum recording unit for PA, participants’ PA levels were categorized into four groups based on their weekly PA duration: inactive (< 30 min), low activity level (30–150 min), moderate activity level (151–300 min), and high activity level (more than 300 min). [22]

### 2.4. Measurement

Trained members of the research team performed all measurements and measured according to the standardized procedure. (1) Height and weight were measured using an HLZ‐63 height–weight analyzer (Tianjin Hualizheng Electronic Technology Co Ltd.). (2) BMD was measured at the calcaneus (heel bone) using the SONOST‐2000 ultrasonic bone densitometer (OsteoSys, Korea). All measurements were performed in strict compliance with SOPs—for example, maintaining consistency in subject posture, probe placement, and coupling medium application. Measurement results were monitored in real time and periodically verified to identify and recheck anomalous data points. (3) Muscle mass was measured using the HBF‐701 body fat analyzer (Omron Healthcare Co Ltd Japan). Absolute muscle mass and relative muscle mass were calculated as TSM and TSMI.

TSM=Muscle Mass×Body WeightTSMI=TSM/Height2

### 2.5. Diagnostic Criteria for Abnormal BMD

BMD is commonly diagnosed using the *T*‐score of BMD. The WHO criteria (1994) were used as the benchmark, *T* ≥ −1.0 is classified as normal BMD, whereas *T* < −1.0 is considered low bone mass or osteoporosis. In this study, due to the rare occurrence of osteoporosis, subjects with BMD < −1.0 were grouped together as abnormal BMD. [23]

### 2.6. Statistical Analysis

**Figure 1:**
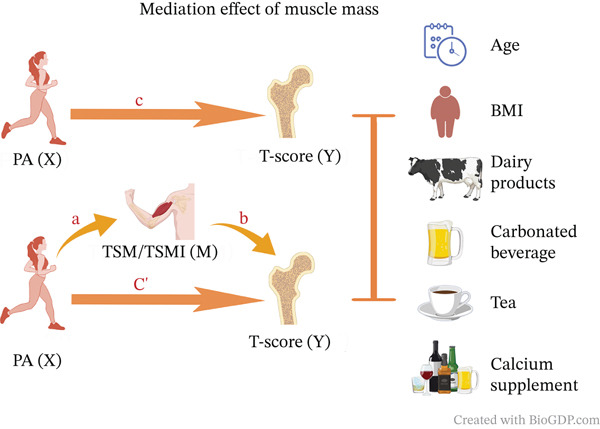
Mediation effect of muscle mass.

Statistical analyses were conducted using R 4.5.1. After data cleaning, we further carried out descriptive statistics and visual analysis. Mean and SD were employed to represent normally distributed continuous variables, and independent *t*‐tests were utilized for group comparisons. Categories were provided as frequencies and percentages (*n* [%]), with chi‐square or Fisher′s exact tests for group comparisons and LSD *t*‐tests for within‐group pairwise comparisons. Both linear regression models and logistic regression models were utilized to investigate the relationships among muscle mass, PA, and calcaneal BMD. Additionally, a mediation model was employed to evaluate the mediating effect of muscle mass between PA and BMD in female university students, [24] with potential confounding factors adjusted as covariates. As depicted in Figure 1, the model specified BMD as the dependent variable (*Y*), PA as the independent variable (*X*), and muscle mass as the mediator (*M*). The mediation model disaggregates the causal effect of *X* on *Y* into an indirect effect through *M* and a direct effect (Path c). The impact of *X* on *M* is shown in Path a, whereas the impact of *M* on *Y* after taking *X* into consideration is shown in Path b, and the mediation process posits that the *X*–*Y* relationship is an indirect effect (Path c) mediated by *M*. [25, 26] To provide robust estimates of mediation effects, both the product‐of‐coefficients method and bootstrap procedures (5000 resamples, 95% confidence intervals) were applied. Subsequently, mediation sensitivity analyses and subgroup analyses were performed.

## 3. Results

### 3.1. BMD, Muscle Mass, and Lifestyle Characteristics in Female University Students

Among the 518 subjects, 210 (40.5%) were in the normal BMD group, whereas 308 (59.5%) were in the abnormal BMD group. More than half of the subjects exhibited abnormal BMD, suggesting a relatively high prevalence of bone density abnormalities among the researched cohort. The mean BMI in the normal group was slightly higher compared with the abnormal group (20.46 ± 2.47 vs. 20.07 ± 2.38), and the difference did not reach statistical significance (*t* = 1.775, *p* = 0.0766 > 0.005). The mean TSM in the normal group was significantly higher than that in the abnormal group (14.90 ± 1.81 vs. 14.39 ± 1.70), with a high level of statistical significance (*t* = 3.216, *p* = 0.0014 < 0.005). Similarly, the mean TSMI in the normal group was significantly higher than that in the abnormal group (5.71 ± 0.55 vs. 5.56 ± 0.50), with a high statistical significance (*t* = 3.304, *p* = 0.0010 < 0.005), further supporting a potential association between higher muscle mass and normal BMD, as shown in Table 1.

**Table 1:** Calcaneal BMD and muscle mass in female university students.

	Normal BMD group	Abnormal BMD group	*t*	*p*
*T*‐value	−0.34 ± 0.48	−1.60 ± 0.39	31.365	0.0000 ^∗∗∗^
BMI (kg/m^2^)	20.46 ± 2.47	20.07 ± 2.38	1.775	0.0766
TSM (kg)	14.90 ± 1.81	14.39 ± 1.70	3.216	0.0014 ^∗∗^
TSMI (kg/m^2^)	5.71 ± 0.55	5.56 ± 0.50	3.304	0.0010 ^∗∗^

^∗^ *p* < 0.05,  ^∗∗^ *p* < 0.01,  ^∗∗∗^ *p* < 0.001.

Chi‐square analysis results showed a statistically significant difference in the rate of abnormal BMD among female university students with different PA (*χ* ^2^ = 9.502, *p* = 0.0233 < 0.05). Over 80% of participants were low‐active (47.5%) or inactive (37.1%), indicating physical inactivity among the subjects. Pairwise comparisons of abnormal BMD rates across different PA showed that, compared with those who are high‐active, the abnormal BMD rates were higher among female university students who are moderate‐active (*p* = 0.040 < 0.05), low‐active (*p* = 0.038 < 0.05), and those who are inactive (*p* = 0.004 < 0.05). Moreover, no statistically significant differences in the rates of abnormal BMD were observed with regard to other lifestyle factors (*p* > 0.05). The results are displayed in Table 2.

**Table 2:** Lifestyle characteristics of female university students.

	Sample size	Normal BMD group *n*(%)	Abnormal BMD group *n*(%)	*χ* ^2^	*p*
Physical activity				9.502	0.0233 ^∗^
High‐active	23	15 (65.2)	8 (34.8)		
Moderate‐active	57	23 (40.4)	34 (59.6)^a^		0.040 ^∗^
Low‐active	246	106 (43.1)	140 (56.9)^a^		0.038 ^∗^
Inactive	192	66 (34.4)	126 (65.6)^a^		0.004 ^∗∗^
Dairy products				1.055	0.7878
Daily	40	18 (45.0)	22 (55.0)		
4–6 times	111	47 (42.3)	64 (57.7)		
1–3 times	205	84 (41.0)	121 (59.0)		
Rarely or never	162	61 (37.7)	101 (62.3)		
Carbonated beverage				1.583^b^	0.6632
Daily	1	1 (100.0)	0 (0.0)		
4–6 times	16	6 (37.5)	10 (62.5)		
1–3 times	151	60 (39.7)	91 (60.3)		
Rarely or never	350	143 (40.9)	207 (59.1)		
Tea				6.831^b^	0.0775
Daily	19^a^	6 (31.6)	13 (68.4)		
4–6 times	18^a^	12 (66.7)	6 (33.3)		
1–3 times	153	56 (36.6)	97 (63.4)		
Rarely or never	328	136 (41.5)	192 (58.5)		
Calcium supplement				0.185	0.6669
Yes	40	18 (45.0)	22 (55.0)		
No	478	192 (40.2)	286 (59.8)		
Total	518	210 (40.5)	308 (59.5)		

^a^Compared with “high‐active” group, *p* < 0.05.

^b^This indicates exact probability test.

^∗^ *p* < 0.05,  ^∗∗^ *p* < 0.01.

### 3.2. Correlation Analyses

Results from linear regression showed a significant positive correlation between muscle mass and calcaneal BMD *T*‐score in both unadjusted and adjusted (*β*_TSM_ = 0.059 and 0.089, *β*_TSMI_ = 0.117 and 0.265, *p* < 0.05), confirming a linear relationship. Additionally, there was a significant positive correlation between PA and BMD in both unadjusted (*β* = −0.104, *p* < 0.05) and adjusted (*β* = −0.109, *p* < 0.05), indicating that higher PA levels were associated with a lower risk of abnormal BMD, as shown in Table 3.

**Table 3:** Multiple linear regression analysis of muscle mass and PA and BMD in female university students.

	Adjusted *R* ^2^	Coefficient	*p*
TSM			
Model 1	0.022	0.059	0.0004 ^∗∗^
Model 2	0.042	0.089	0.0000 ^∗∗^
TSMI			
Model 1	0.007	0.117	0.0355 ^∗^
Model 2	0.025	0.265	0.0040 ^∗∗^
PA			
Model 1	0.011	−0.104	0.0183 ^∗^
Model 2	0.044	−0.109	0.0169 ^∗^

*Note: Model 1 does not adjust for any factors; Model 2 adjusts for age, BMI, intake of dairy products, carbonated beverage, tea, and calcium supplement.*

^∗^p < 0.05,  ^∗∗^p < 0.01.

To further investigate the association, logistic regression analysis was conducted. The results showed that both absolute muscle mass (TSM) and relative muscle mass (TSMI) exhibited negative coefficients in both unadjusted and adjusted (*β*_TSM_ = −0.139 and −0.193, *β*_TSMI_ = −0.343 and −0.682, *p* < 0.05), demonstrating an inverse association between muscle mass and the probability of abnormal BMD, and showed that PA serves as a protective factor against abnormal BMD in female university students in both unadjusted and adjusted (*β* = 0.293 and 0.310, *p* < 0.01), as displayed in Table 4.

**Table 4:** Logistic regression analysis of muscle mass, PA, and BMD in female university students.

	*β*	SE	Wald*χ* ^2^	*p*	OR (95% CI)
TSM					
Model 1	−0.139	0.045	9.564	0.00198 ^∗∗^	0.870 (0.795, 0.949)
Model 2	−0.193	0.060	10.312	0.00132 ^∗∗^	0.825 (0.731, 0.925)
TSMI					
Model 1	−0.343	0.146	5.528	0.01870 ^∗^	0.709 (0.530, 0.941)
Model 2	−0.682	0.261	6.806	0.00910 ^∗∗^	0.506 (0.296, 0.829)
PA					
Model 1	0.293	0.113	6.753	0.00936 ^∗∗^	1.340 (1.073, 1.674)
Model 2	0.310	0.119	6.876	0.00874 ^∗∗^	1.364 (1.080, 1.722)

*Note:* Model 1 does not adjust for any factors; Model 2 adjusts for age, BMI, intake of dairy products, carbonated beverage, tea, and calcium supplement.

^∗^ *p* < 0.05,  ^∗∗^ *p* < 0.01.

### 3.3. Mediation Analysis, Sensitivity Analysis and Subgroup Analysis

Mediation effect analyses and sensitivity analyses were reconducted to explore potential mediating pathways after data cleaning. PA was significantly negatively correlated with both TSM and TSMI in both BMI unadjusted and BMI adjusted (all total effect coefficients ranged from −0.099 to −0.103, *p* < 0.01). The proportion of mediation (prop. mediated) by muscle mass was substantial (78.64%–79.21% for TSM and 85.86%–86.28% for TSMI). Mediation effects were significant in TSMI models in both BMI unadjusted and BMI adjusted (ACME = −0.088 and −0.085, CI excluding 0), whereas those in TSM models were only marginally significant (ACME = −0.081 and −0.080, CI excluding 0). Sensitivity analyses revealed consistent thresholds of Rho = 0.1, indicating that the mediation effects were sensitive to weak unobserved confounders. After adjusting for BMI, the mediation effect of TSMI was more sensitive to unobserved confounders, warranting attention to potential confounding factors. The results are displayed in Table 5.

**Table 5:** Mediation and sensitivity analysis of muscle mass in the relationship of PA and BMD.

	Total effect	ACME	ACME 95% CI	Prop. mediated	Rho	*R* ^2^_*M* *R* ^2^_*Y*
TSM						
BMI unadjusted	−0.103 ^∗∗^	−0.081 ^∗^	(−0.170, 0.001)	78.641%	0.1	0.01
BMI adjusted	−0.101 ^∗∗^	−0.080∗	(−0.170, 0.003)	79.208%	0.1	0.01
TSMI						
BMI unadjusted	−0.102 ^∗∗^	−0.088 ^∗∗^	(−0.178, −0.004)	86.275%	0.1	0.01
BMI adjusted	−0.099 ^∗∗^	−0.085 ^∗∗^	(−0.174, −0.002)	85.859%	0.1	0.01

^∗^Mediation effect marginally significant (CI approaching 0) and  ^∗∗^mediation effect significant (CI excluding 0).

**Figure 2:**
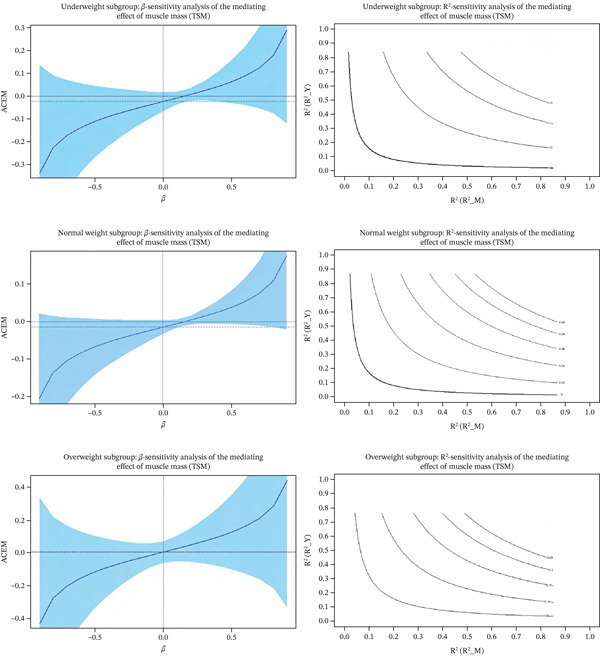
Sensitivity analysis of the mediating effect of the muscle mass (TSM).

**Figure 3:**
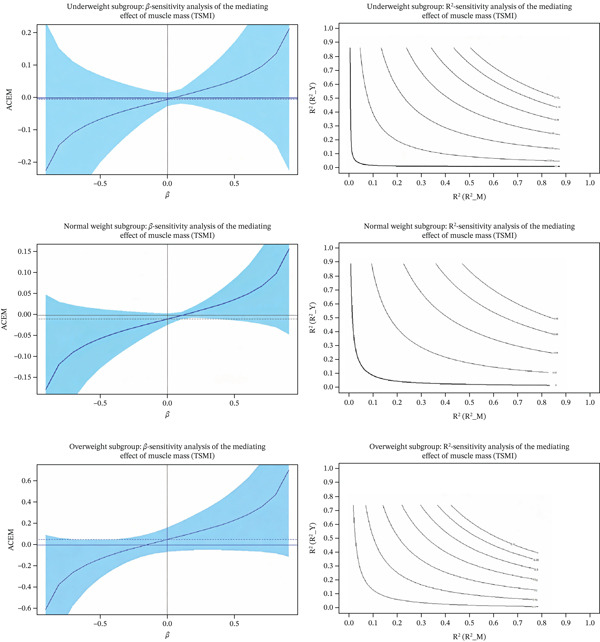
Sensitivity analysis of the mediating effect of the muscle mass (TSMI).

Given that BMI might act as a confounder, further subgroup and mediation sensitivity analyses were conducted. BMI was categorized according to the WHO Asian criteria into underweight (BMI < 18.5), normal weight (18.5 ≤ BMI < 24), and overweight (BMI ≥ 24). The results showed that only the overweight group exhibited significant mediation effects (ACME = −0.468, CI excluding 0), whereas the mediation effects in the normal weight and underweight groups were nonsignificant. Sensitivity analyses indicated that the mediation effect of TSMI in the overweight group had relatively high robustness (Rho = −0.2, R^2^_MR^2^_Y = 0.04), whereas other subgroups showed varying degrees of sensitivity to unobserved confounders, as shown in Table 6. Figure 2 and Figure 3 showed the sensitivity analysis of the mediating effect of the muscle mass.

**Table 6:** Mediation sensitivity analysis and BMI subgroup analysis of muscle mass in the relationship of PA and BMD.

	Total effect	ACME	ACME 95% CI	Prop. mediated	Rho	*R* ^2^_*M* *R* ^2^_*Y*
TSM						
Normal weight	−0.064	−0.078	(−0.168, 0.033)	121.875%	0.1	0.01
Underweight	−0.074	−0.097	(−0.295, 0.179)	131.081%	0.2	0.04
Overweight	−0.420 ^∗∗^	−0.468 ^∗∗^	(−0.896, −0.123)	99.524%	0	0
TSMI						
Normal weight	−0.078	−0.070	(−0.173, 0.030)	89.744%	0.1	0.01
Underweight	−0.097	−0.093	(−0.319, 0.171)	95.876%	0	0
Overweight	−0.418 ^∗∗^	−0.468 ^∗∗^	(−0.900, −0.185)	111.962%	−0.2	0.04

*Note:* Models were adjusted for age, intake of dairy products, carbonated beverage, tea, and calcium supplement. Prop. mediated describes the proportion of mediation effect in the total effect.

^∗∗^indicates statistical significance.

## 4. Discussion

The female population is a high‐risk demographic for osteoporosis, with a meta‐analysis indicating that the incidence of osteoporosis among Chinese women post‐20 years of menopause reaches 78.3%. [27] The university years are essential for physical development and bone mass accumulation, whereas peak muscle mass in adulthood is crucial for sustaining bone mass and strength. [28] Interventions at this period can significantly augment peak bone mass, establishing a solid foundation for enduring skeletal health. Nonetheless, a large percentage of university students have decreased bone mass or even osteopenia, which is a sign of suboptimal bone health. [29, 30]

Although some studies have begun to investigate the relationship among PA muscle mass, and BMD, the majority of research is restricted to hospital‐based populations and postmenopausal women, [31–33] with few studies conducted among female university students, particularly the quantitative analysis of the mediating mechanism, which urgently requires improvement.

### 4.1. Effect of Muscle Mass on BMD

The mediating effect of muscle mass between PA and BMD can be primarily explained from a biomechanical and endocrine perspective. Firstly, muscle, bone, and PA form a closely integrated functional unit, and their interactions constitute the core mechanism for maintaining skeletal health. [34] The basic theoretical framework is that PA, especially weight‐bearing and high‐impact exercise, promotes bone health through two main pathways: directly applying mechanical stress to bones, and increasing muscle mass and strength, which in turn generates greater tension on bone attachment points through muscle contraction. This process is based on Frost′s “mechanostat theory”, which states that bone tissue can sense the mechanical strain it endures; when the strain exceeds a specific threshold, it activates bone formation to adapt to the new load environment. [35] Muscle mass plays a key mediating role in this process. [36] PA stimulates muscle growth and strength enhancement, and larger, stronger muscles can generate more efficient mechanical stimulation to bones, thereby promoting increased BMD. Proctor′s study revealed that muscle mass is among the strongest predictors of BMD, accounting for 6%–53% of BMD variance. [37]

Beyond pure biomechanical effects, muscle, as an endocrine organ, can secrete various myokines, cytokines synthesized by myocytes upon contraction signal stimulation. These factors may directly regulate osteocyte activity via paracrine or endocrine pathways, providing another crucial biochemical pathway for muscle–bone crosstalk. [38]

Myokines modulate bone metabolism through various signaling pathways, like the Wnt and MAPK signaling cascades. In the regulation of osteoblast differentiation and bone formation, the Wnt/*β*–catenin pathway is of paramount importance. [39] Wnt signaling controls critical biological processes such as stem cell differentiation and the activity of osteoblasts and osteoclasts, vital for maintaining bone mass and integrity. [40] Muscle‐derived substances and electrical stimulation can activate molecules associated with the Wnt pathway to improve signal transduction [41]. Conversely, substances and muscle absence inhibit important pathway molecules, disrupt signal transduction, and reduce the osteogenic activity of bone cells. MAPK signaling is indispensable for bone development. Virtually all extracellular ligands found to affect bone biology function, at least partially, via MAPK pathways. [42] The MAPK pathway functions in concert with several signaling pathways, demonstrating intricate connections. For example, TAK1, which belongs to the MAPKKK family, is involved in the regulation of TGF‐*β* signal transduction. [43]

Recent studies have elucidated the paracrine regulatory function of myokines in the interaction between bones and muscles [44, 45]. Myostatin, a constituent of the TGF‐*β* superfamily, functions significantly as an inhibitor of skeletal muscle growth [46] has a multifaceted role in bone production [44] and resorption [45] and modulates bone cell activity via many methods, including the Wnt/*β*–catenin signaling pathway [39] and the BMP signaling system. [47] Irisin, a factor induced by PA, is pivotal in bone metabolism and the development of osteoporosis. It promotes osteoblast proliferation, differentiation, and mineralization primarily via the MAPK, ERK, and AMPK signaling pathways, while modulating osteoclast differentiation and maturation through the Wnt/*β*–catenin, JNK, and RANKL/RANK/OPG signaling pathways [48] In addition to myostatin and irisin, other myokines such as interleukin‐6 (IL‐6) [49] and IL‐15 [50] also participate in the paracrine regulation of bone metabolism.

Apart from the two extensively investigated mechanisms, the mediating role of muscle mass can further be realized via the following pathways.

Increased muscle mass promotes the synthesis of sex hormones such as testosterone and estrogen; they not only directly enhance osteoblast activity but also effectively inhibit bone resorption. For instance, insufficient testosterone levels lead to an elevated RANKL/OPG ratio in the body, thereby accelerating the differentiation and maturation of osteoclast. [51]

Regular PA maintains skeletal muscle mass, which serves as the principal reservoir for antioxidant enzymes (e.g., SOD and GPx). [52] Augmented muscle mass enhances systemic antioxidant capacity, effectively scavenging excessive reactive oxygen species (ROS), which if accumulated excessively, would impede osteoblast differentiation and enhance osteoclastogenesis. [53] Conversely, muscle wasting (e.g., resulting from physical inactivity) compromises antioxidant defense mechanisms, exacerbating oxidative stress, which in turn accelerates muscle degradation and a decline in BMD, forming a vicious cycle. [54] Notably, ROS exhibits biphasic effects: Acute, moderate ROS generation triggered by exercise serves as a signaling molecule to facilitate myocyte hypertrophy and osteogenesis, whereas chronic overproduction perturbs the homeostasis of the musculoskeletal system. [53]

Regular PA helps maintain muscle mass, which in turn reduces the release of pro‐inflammatory cytokines such as tumor necrosis factor‐*α* (TNF‐*α*) and IL‐6. [55] If these cytokines accumulate excessively in the body, they activate the NF‐*κ*B signaling pathway, which not only inhibits osteoblast proliferation and promotes osteoclast activation but also accelerates muscle protein degradation via the ubiquitin–proteasome system. This creates a destructive cycle that exacerbates both sarcopenia and osteoporosis. [55]

Sufficient muscle mass enhances the uptake and utilization of nutrients like protein, calcium, and phosphorus. In contrast, protein deficiency disrupts the balance of muscle protein metabolism, reduces intestinal calcium absorption, and ultimately impairs the synthesis of bone matrix. [56]

These mechanisms have been progressively validated and refined in recent studies focusing on musculoskeletal metabolism.

### 4.2. Moderating Role of BMI: Enhanced Mediating Effect in the Overweight Group

The study found that the mediating effect of muscle mass showed poor robustness in the overall population of female university students, a common phenomenon in related studies, possibly due to the model′s failure to fully capture complex biological factors. Bone health is influenced by confounding factors such as genetics, hormone levels, and intake of multiple nutrients, and unmeasured or incompletely controlled variables may weaken the stability of the mediating effect. Moreover, measurement limitations of the PA self‐assessment questionnaire may introduce errors affecting result robustness.

The most insightful finding is that after subgroup analysis by BMI, the mediating effect in the overweight group was significant with relatively high robustness. This result suggests that BMI may play a key regulatory role in muscle–bone interactions, rather than merely serving as a confounding factor to be controlled.

Compared with normal‐weight individuals, overweight individuals experience higher static and dynamic mechanical loads on bones during daily activities due to greater body weight. [57] A study on elderly women showed that body weight explained much more of bone mass variation (15%–32%) than PA or muscle strength (1%–6%), strongly supporting the fundamental role of weight loading. [57] This persistently high basal load may keep bones in a “preactivated” state long‐term. During additional PA, the extra stress from muscle contraction can more easily trigger adaptive bone remodeling. In other words, in overweight individuals, the “signal” from PA to bones via muscle mass may be amplified, making the mediating pathway clearer and more robust. [58–60]

Additionally, the underlying mechanisms also include the threshold effect of bone adaptation: Bone growth and maintenance require a specific threshold of mechanical stimulation. Overweight individuals′ daily load may be close to or exceed this threshold, thus the extra stimulation from increased muscle mass driven by PA can more easily produce significant bone gain. [61, 62] In contrast, normal or underweight individuals may need higher intensity of PA to achieve equivalent stimulation, resulting in unstable effects at moderate intensity.

Moreover, overweight not only means higher weight but also usually higher lean mass and fat mass. Although excessive fat is detrimental to overall health, moderate fat can protect bones through multiple pathways. For example, adipokines like leptin secreted by adipose tissue can promote bone formation. [63] Thus, in overweight individuals, higher muscle mass and fat mass may synergistically provide a more favorable physiological environment for bone health, enhancing the mediating effect.

### 4.3. Analysis of Differences Among Different Populations

Higgins′s research indicates that gender disparities exist in the mediating effect of muscle, with muscle mass and muscle strength exhibiting distinct functions in the impact of PA on bone among young adults. [64] The mediating effect of muscle can be influenced by various factors, including estrogen levels, exercise types and intensity, and individual variations. Playing a critical role in the muscle–bone crosstalk, [65] estrogen serves as an important regulator. [66] For instance, estrogen regulates sclerostin expression induced by bone morphogenetic proteins via the Wnt signaling pathway. [67] According to Zymbal et al.′s study, PA interventions in growing individuals that increase muscle mass are effective for both males and females throughout childhood and adolescence, especially in females. [68] For female university students, whether majoring in sports or not, PA can modify lean body weight and improve muscle strength. Nevertheless, in the absence of specialized strength training, the overall changes in muscle strength remain relatively slight [69] which may not meet the mechanical threshold required for bone remodeling.

Consistent studies have shown that PA positively affects BMD and muscle mass across different age groups in women, with muscle mass playing a significant role. A study on women aged 16–20 revealed that PA levels were positively correlated with BMD at the lumbar spine and forearm, with lean body mass and muscle strength being important predictors of BMD. [70] Another study indicated a positive correlation between muscle strength and BMD in young women (18–31 years old) [71] A cross‐sectional study on women aged 38–64 found that regularly exercising women had significantly higher BMC at the distal third of the radius compared with the control group. [72] A study on Italian middle‐aged women also confirmed a positive association between PA and lumbar spine BMD. [73] Postmenopausal women experience accelerated bone loss and increased risk of osteoporosis due to decreased estrogen levels. [74] At this stage, PA plays a more critical role in slowing bone loss. [75] According to Xiang et al.′s study, lean mass served as a mediator in the association between PA and BMD in postmenopausal women. [76] In postmenopausal women, the mediating role of muscle may be more important due to declining estrogen levels, whereas in female university students with relatively stable estrogen levels, muscle development and function are likely more critical for peak bone mass accumulation. The findings provide meaningful insights for preventing bone mass loss in women.

The lifestyle characteristics of Chinese female university students (such as prolonged sedentary time and insufficient PA) differ significantly from their foreign peers of the same age, and the mechanism by which muscle mass acts on bone health shows uniqueness. The PA pattern of Chinese female university students is predominantly low‐intensity. A study published in the Journal of Sichuan University found that female university students mainly engaged in low intensity PA. Moderate intensity and vigorous intensity were positively correlated with BMD, whereas sedentary time was negatively correlated with it. [77] Both domestic and international studies have confirmed that sedentary lifestyle is a well‐established unhealthy lifestyle risk factor for osteoporosis [78–79] In contrast, female university students in European and American countries generally have higher levels of PA. [80]

Both domestic and international studies have shown that muscle mass plays a mediating role in the association between PA and BMD. A follow‐up study of 1200 adolescents in Valparaiso from the foreign Cogni‐Action project showed that the ASMI can explain 29.7% of the negative correlation between fat and bone mineral content (BMC), with the mediating effect in females (32.9%) being significantly higher than in males. [81]

### 4.4. Practical Implications for Bone Health Interventions

The above studies indicate that even the same intervention may yield different effects in different populations. For female university students, bone health education should be strengthened, with emphasis on the importance of BMD during adolescence and the impacts of PA and balanced nutrition on skeletal health. [82] In particular, it is necessary to correct unhealthy weight management concepts such as excessive dieting, and encourage female university students to engage in weight‐bearing exercises such as rope skipping, running, basketball, and volleyball, as well as resistance training like squats and lunges. [83, 84] Given academic pressure, female university students can be encouraged to engage in fragmented exercises such as walking and taking stairs during breaks and commuting time to reduce prolonged sitting and accumulate PA. Increasing consumption of calcium‐rich foods such as dairy products, soy products, and green leafy vegetables is recommended. [85] Meanwhile, pay attention to vitamin D supplementation, which can promote calcium absorption through sun exposure or supplements. [86] In addition to exercise and nutrition, other lifestyle factors such as sleep and stress management should also be considered, as they may indirectly affect bone health. [87]

Develop personalized exercise and nutrition plans for individuals with different BMI levels. For overweight individuals, emphasis can be placed on resistance training and improvement of muscle mass to achieve more stable improvement in BMD. When promoting bone health interventions in different countries and regions, it is necessary to consider the physical characteristics, lifestyles, and sociocultural backgrounds of the local population. For example, in countries with a high obesity rate, bone health interventions for overweight populations may need to focus more on muscle training and weight management. In regions where calcium intake is insufficient due to traditional dietary habits, strengthening dietary nutrition guidance is more critical.

This study has certain limitations. For example, the study used the quantitative ultrasound method (QUS) to assess the *T*‐score and determine whether subjects had abnormal BMD. Although the DXA (dual‐energy x‐ray absorptiometry) method is internationally recognized as the gold standard for diagnosing osteoporosis based on area BMD measurements, [88] the QUS measuring instrument is easy to operate, free from radiation, more portable and cost‐effective, with results showing good correlation with the DXA measurement, [89] making them appropriate for extensive screening and applicable in settings with limited resources. Numerous studies, both domestic and international, have utilized QUS to measure BMD. [90–91] QUS has limitations such as technical variations, standardization issues, and incomplete consistency with DXA results. Subsequent investigations need to concentrate on developing standardized QUS measurement protocols and further evaluate QUS′s predictive capacity for fracture risk in adolescents.

In conclusion, this study provides new evidence for the formulation of bone health intervention strategies by in‐depth analysis of the mediating role of muscle mass in the relationship between PA and BMD, combined with the analysis of the specific background of Chinese female university students. Future studies should further expand the scope of research subjects and adopt more sophisticated research designs to comprehensively reveal the complex interaction mechanisms between PA, muscle mass, and BMD.

## 5. Conclusion

The results of this investigation showed a strong positive relationship among muscle mass, PA, and calcaneal BMD in female university students. Specifically, higher levels of muscle mass and PA were associated with increased calcaneal BMD values, indicating that high activity level serves as a protective factor against abnormal BMD. Subgroup and mediation sensitivity analyses indicated that the mediation effect only in female university students of the overweight group had significant mediation effects and high robustness. This suggests that the beneficial impact of PA is primarily achieved through the enhancement of muscle mass, highlighting the critical role of muscular development in maintaining optimal bone health during this developmental stage.

## Author Contributions

(1) Jiaye Wang, Zheqing Zhang, Xiaocui Lu, and Yiran Yan conceived and designed the study; (2) Jiaye Wang, Zheqing Zhang, Shitian Li, Huanyu Hu, Haojian Chen, and Zhendi Zhang collected the data; (3) Jiaye Wang, Zheqing Zhang, Shitian Li, Huanyu Hu, Haojian Chen, and Zhendi Zhang analyzed and interpreted the data; (4) Jiaye Wang and Shitian Li wrote the manuscript; (5) Xiaocui Lu and Yiran Yan provided critical revisions that are important for the intellectual content; (6) Jiaye Wang and Zheqing Zhang contributed equally to this work.

## Funding

This study was supported by the National Natural Science Foundation of China (10.13039/501100001809) (82373559) and Southern Medical University Student Innovation and Entrepreneurship Training Program (201812121226).

## Disclosure

All authors approved the final version of the manuscript. They take full responsibility for the integrity and accuracy of the publication.

## Ethics Statement

The Biomedical Ethics Committee of Southern Medical University granted approval for this investigation (approval number: Medical Ethics Review of Southern Medical University [2022] No. 32), and all research adhered to the moral guidelines for research with human subjects established by the World Medical Association Declaration of Helsinki. Written informed consent was obtained from the participants.

## Consent

Written informed consent was obtained from the participants.

## Conflicts of Interest

The authors declare no conflicts of interest.

## Data Availability

The datasets used and/or analyzed during the current study are available from the corresponding authors on reasonable request.
